# The utility of urinary CD80 as a diagnostic marker in patients with renal diseases

**DOI:** 10.1038/s41598-018-35798-2

**Published:** 2018-11-23

**Authors:** Shogo Minamikawa, Kandai Nozu, Shingo Maeta, Tomohiko Yamamura, Keita Nakanishi, Junya Fujimura, Tomoko Horinouchi, China Nagano, Nana Sakakibara, Hiroaki Nagase, Hideaki Shima, Kenta Noda, Takeshi Ninchoji, Hiroshi Kaito, Kazumoto Iijima

**Affiliations:** 1 Department of Pediatrics, Kobe University Graduate School of Medicine, Kobe, Japan; 2 Sysmex Corporation, Technology Development, Elemental Technology Development 2, Kobe, Japan

## Abstract

CD80, which regulates T cell activation, may provide a differential diagnostic marker between minimal change disease (MCD) and other renal diseases, including focal segmental glomerular sclerosis (FSGS). However, recent reports show contrasting results. Therefore, we evaluated the utility of urinary CD80 as a diagnostic biomarker. We collected 65 urine samples from 55 patients with MCD (n = 31), FSGS (n = 4), inherited nephrotic syndrome (n = 4), Alport syndrome (n = 5) and other glomerular diseases (n = 11), and control samples (n = 30). We measured urinary CD80 levels by ELISA. Urinary CD80 (ng/gCr) (median, interquartile range) levels were significantly higher in patients with MCD in relapse (91.5, 31.1–356.0), FSGS (376.2, 62.7–1916.0), and inherited nephrotic syndrome (220.1, 62.9–865.3), than in patients with MCD in remission (29.5, 21.7–52.8) (p < 0.05). Elevation of urinary CD80 was observed, even in patients with inherited nephrotic syndrome unrelated to T cell activation. Additionally, urinary CD80 was positively correlated with urinary protein levels. Our results suggest that urinary CD80 is unreliable as a differential diagnostic marker between MCD in relapse and FSGS or inherited kidney diseases. Increased urinary CD80 excretion was present in all patients with active kidney disease.

## Introduction

Nephrotic syndrome (NS) is clinically characterized by massive proteinuria with hypoalbuminemia, accompanied by systemic oedema. These clinical changes are correlated with specific structural changes in the foot processes of glomerular visceral epithelial cells, podocytes, which form the glomerular filtration barrier^1,2^. NS is categorized into primary diseases of idiopathic NS, such as Minimal Change Disease (MCD) and Focal Glomerulosclerosis (FSGS), and secondary diseases associated with drugs, infection or genetic defects, i.e., inherited NS^3^.

In MCD, the most common cause of NS in children, the activation of T cells may be related to injuries of the glomerular filtration barrier, including podocytes, for the following reasons:^4^ (i) remission of MCD is caused by diseases and drugs leading to the inactivation of T cells, such as measles, steroids, and cyclosporine; (ii) Hodgkin’s diseases associated with T-cell activation often cause secondary MCD.

However, in inherited NS, variants of podocyte-related genes are linked, directly or indirectly, to specific defects of podocytes^5^; most patients with deleterious variants of these genes show significantly lower response to immunosuppressive therapy, or entirely lack a response to such therapy^6,7^. This suggests that inherited NS is not associated with T-cell activation.

CD80 is a transmembrane protein which is expressed on antigen-presenting cells or natural killer cells. It works as a ligand, playing important roles in T-cell activation and inactivation by binding to CD28 on T cells or cytotoxic T-lymphocyte-associated-4 (CTLA-4) on Tregs^8^. Gene expression of CD80 is upregulated by allergens and irritants in human keratinocytes, or by oxidative stress in mastocytoma tumour cells^9,10^. Recently, some groups reported CD80 expression in glomerular podocytes in MCD patients in relapse, as well as in podocytes cultured with serum in the relapse phase of MCD. In addition, CD80 was not present in podocytes from patients with MCD in remission, or patients with FSGS^11,12^. Notably, urinary CD80 was elevated in patients with MCD in relapse, but not in patients in remission or with FSGS^13^. From these results, it was hypothesized that the level of urinary CD80 was useful as a biomarker to distinguish between MCD patients in relapse and other renal diseases, including FSGS^13^.

However, more recent reports have shown different results for the expression of CD80 on podocytes. Reiser *et al*. reported that CD80 was expressed on podocytes in patients with various diseases: MCD in relapse, FSGS, SLE, and even inherited diseases^14^. In contrast to this report, Larsen *et al*. and Novelli *et al*. reported that CD80 was not found on podocytes in either MCD in relapse or FSGS^15,16^. Thus, the specificity of expression of CD80 on podocytes in MCD remains controversial^15,17^. Moreover, the usefulness of urinary CD80 levels as a differential diagnostic marker for patients with MCD has not been fully examined.

The present study was performed to determine the utility of urinary CD80 measurement for the differential diagnosis of patients with various renal diseases. We recruited patients with MCD, FSGS, and other glomerular diseases, including inherited renal diseases that are not associated with T-cell activation.

## Results

### Patient characteristics

The clinical characteristics of each group are shown in Table 1. There were statistically significant differences in urinary protein/creatinine between MCD in remission and MCD in relapse, FSGS, or inherited NS.

**Table 1:** Characteristics of patients with each renal disease.

	MCD in remission	MCD in relapse	FSGS	Inherited NS	Alport syndrome	Other glomerular diseases
Cases (n)	17	24	4	4	5	11
Sample (n)	17	24	4	4	5	11
Ages (year)	11.0 (4.0–14.5)	11.5 (5.8–15.8)	1.5 (1.0–2.0)	9.0 (2.3–11.3)	12.5 (3.0–29.5)	12.0 (9.0–14.0)
Gender (male:female)	11:6	17:7	2:2	4:0	5:0	5:6
Serum Albumin (g/dl)	3.6 (2.7–4.0)	2.7 (1.6–3.7)	2.8 (2.5–2.8)	2.4 (1.8–3.9)	3.1 (1.9–4.1)	3.7 (2.2–3.9)
Proteinuria (g/gCr)	0.3 (0.2–0.7)	10.9^*^ (3.4–17.7)	19.0^*^ (4.4–168.9)	8.2^*^ (2.1–86.4)	0.7 (0.2–4.8)	1.2 (0.6–4.0)

Dunn’s multiple test (vs MCD in remission), all values are median (interquartile range).

*p < 0.05.

### Precision of our ELISA

We produced a standard curve for CD80 with commercially quantified CD80 protein (Cat. No. 10698-H08H, Sino Biological Inc.) (Supplemental Fig. 1). In addition, we also produced a standard curve with an ELISA kit (Bender MedSystems, Burlingame, CA, USA) used in previous reports^13,18^, as a comparison with our ELISA method. Both ELISAs displayed a linear correlation between CD80 concentration and OD450; the correlation coefficient of our ELISA was higher than that of the established ELISA kit.

### Urinary CD80 excretion

**Figure 1:**
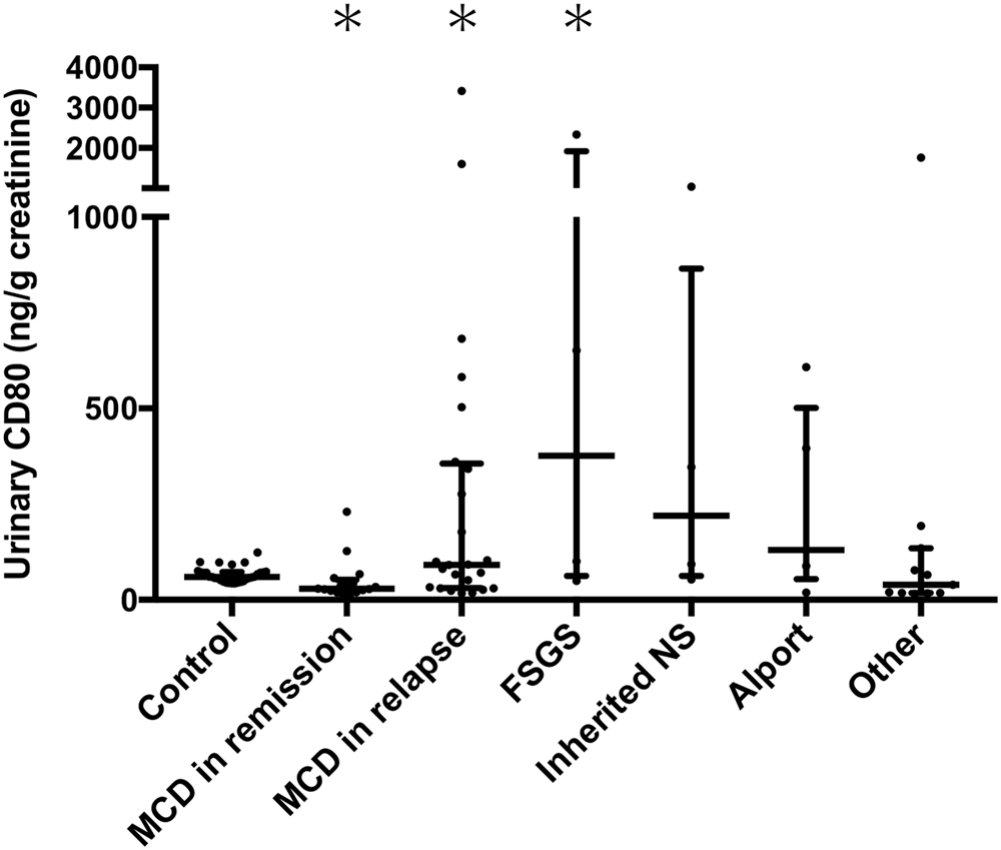
Concentration of urinary CD80 in controls and patients with MCD, FSGS, inherited NS, Alport syndrome, and other glomerular diseases. Patients with MCD in relapse (p = 0.005), FSGS (p = 0.018), and inherited NS (p = 0.032) showed higher urinary CD80 levels than patients with MCD in remission.

The CD80 (ng/gCr) levels in urine were significantly higher in patients with MCD in relapse (91.5, 31.1–356.0), FSGS (376.2, 62.7–1916.0) or inherited NS (220.1, 62.9–865.3) than in patients with MCD in remission (29.5, 21.7–52.8) (p < 0.05) (Fig. 1). Two of four patients with FSGS and one of four patients with inherited NS showed much higher urinary CD80 than 75th percentile of CD80 in patients with MCD in relapse (2337.6, 651.5, and 1038.1 ng/gCr, respectively). There was no significant difference in urinary CD80 level between patients with MCD in remission and controls (59.9, 48.6–72.5). There were no significant differences in urinary CD80 levels among patients with MCD in relapse, FSGS, or inherited NS. To investigate whether elevated urinary CD80 in MCD in relapse has specificity for other renal diseases, we compared urinary CD80 values between MCD in relapse and all renal diseases other than MCD. Notably, no differences in urinary CD80 were found between MCD in relapse and other renal diseases (Supplemental Fig. 2).

### Correlation between CD80 values in serum and urine

**Figure 2:**
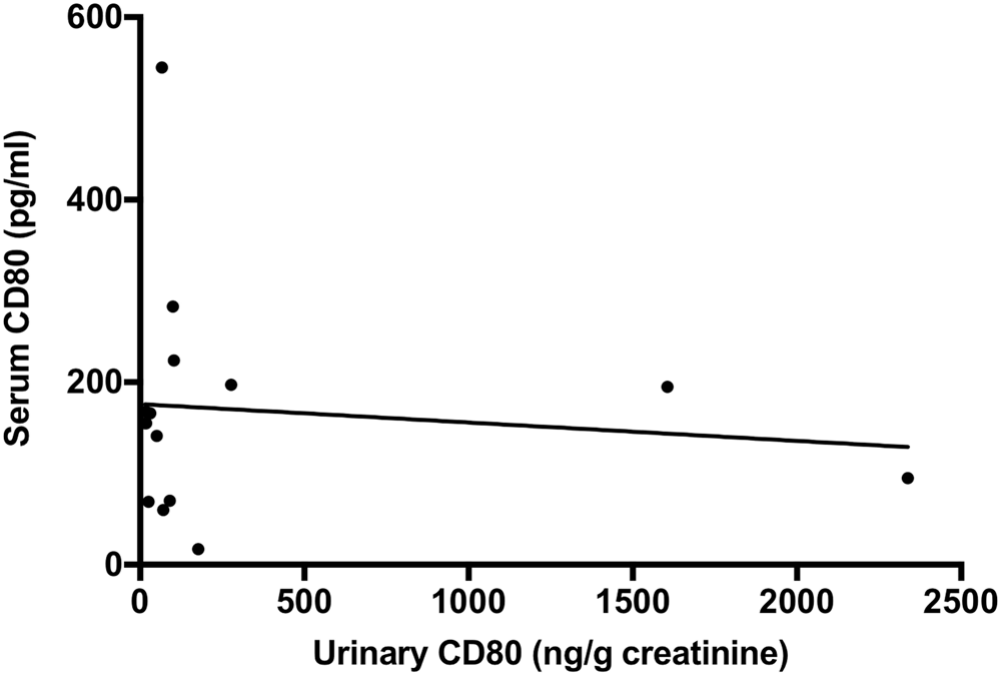
Correlation between serum and urinary CD80. Serum and urine samples collected at the same time points were measured in 15 patients, including 11 with MCD in relapse, one with FSGS, and three with other renal diseases. Most samples remained in the normal range (155, 70–197 pg/ml); no correlation was found between serum and urinary CD80 (r = 0.07, p = 0.80).

The relationship between serum CD80 and urinary CD80 was examined in 15 patients, but no correlation was found (r = 0.07, p = 0.80) (Fig. 2). Serum CD80 remained at a normal value (20–320 pg/ml)^19^, even in patients who exhibited high values, such as urinary CD80 exceeding 1000 ng/gCr.

### Correlation between urinary CD80 and urinary protein

**Figure 3:**
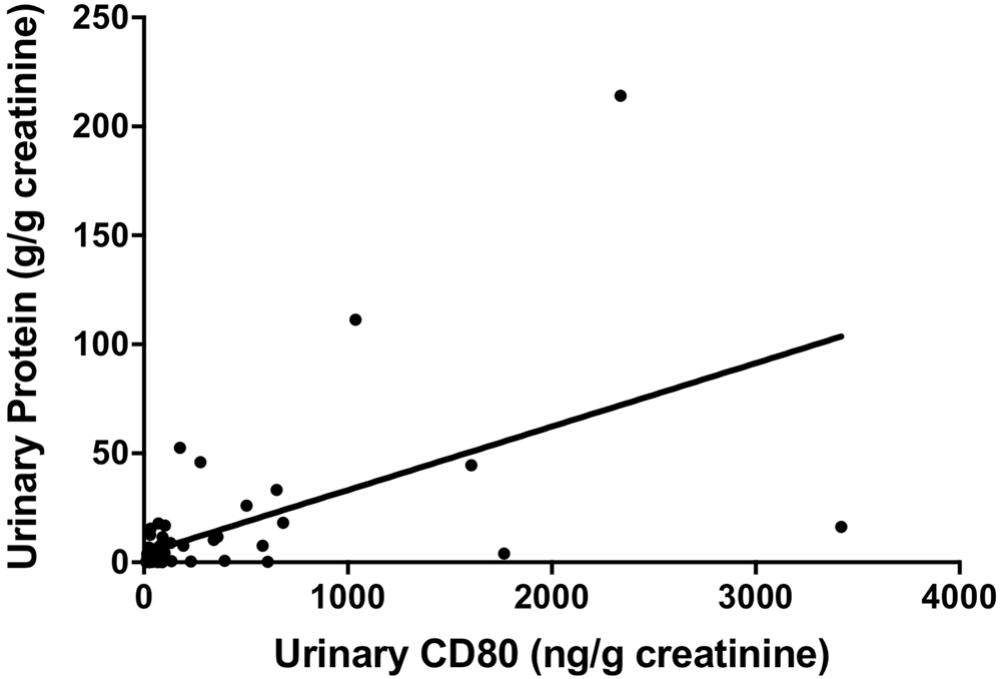
Correlation between urinary CD80 and the degree of proteinuria in total subjects. A positive correlation was found between them (r = 0.57, p < 0.0001).

A positive correlation was found between urinary CD80 levels and the urine protein/creatinine ratio in total subjects (r = 0.57, p < 0.0001) (Fig. 3).

In each group, there were wide variations in urinary CD80 concentration, reflected by wide error bars (Fig. 1). Therefore, we studied the correlation between urinary CD80 and urinary protein in each group. First, we calculated the 97.5th percentile of the control group and determined that the normal range of urinary CD80 lies below 105.1 ng/gCr. Values greater than the 97.5th percentile of control were defined as high. The remaining subjects in each group were defined to exhibit normal CD80 levels. Then, we compared the amounts of urinary protein in these two groups, according to each renal disease. There were significant differences between the high and low groups in MCD in relapse, and for the group of all renal diseases (Supplemental Fig. 2). Although the same tendency was observed between inherited NS and FSGS, a significant difference was not detected because of the small sample size.

## Discussion

In this study, we evaluated the concentration of urinary CD80 of 65 samples from 55 patients with various renal diseases, including inherited diseases, to determine the utility of urinary CD80 as a diagnostic biomarker. Among these 65 samples, 20 were collected from 10 patients at different two points of NS (remission and relapse). The remaining 45 samples were collected from different patients.

Since Garin *et al*. reported the utility of urinary CD80 as a differential biomarker between the relapse phase of MCD and other renal diseases in 2009^13^, five other reports have been published concerning the use of this biomarker^12,18,20–22^. All these reports showed elevation of urinary CD80 only in patients with MCD in relapse, with levels remaining normal in controls, patients with MCD in remission, FSGS or other renal diseases. Specific elevation of urinary CD80 in MCD in relapse was supported by the following reports: (1) The expression of CD80 on podocytes was strongly enhanced in MCD patients in relapse, measured by immunostaining, compared with patients with MCD in remission or those with FSGS^12^. (2) The source of urinary CD80 is the podocytes because the molecular weight of urinary CD80 (53 kDa) is close to that of CD80 derived from cell membrane, rather than that of serum CD80 (23 kDa)^12^. (3) The serum level of CD80 is normal in MCD in relapse although it is elevated in urine^13^. However, some other studies have recently reported that the immunostaining assay of CD80 on podocytes is not a reliable diagnostic marker for MCD in relapse^15,16^.

Although previous reports maintain that urinary CD80 levels clearly differentiate between MCD in relapse and FSGS^12,13,18,23^, our study did not (Fig. 1) (Supplemental Fig. 2). There was some uncertainty, in that previous studies involved FSGS cases derived from genetic defects, whereas our cases with FSGS were derived from immunological mechanisms. To remove this concern, we measured urinary CD80 levels in patients with inherited NS; these patients also exhibited elevated CD80. This result supports our conclusion that urinary CD80 is not a reliable marker for MCD in relapse.

In our study, the concentration of CD80 in urine was higher in patients with FSGS and inherited NS in addition to MCD in relapse than MCD in remission. This might mean that excretion of urinary CD80 may increase in patients with active renal disease, rather than upon activation of T cells. CD80 has been previously measured in patients with inherited renal diseases^14,23^. Cara-Fuentes *et al*. reported a patient with an *NPHS2* defect who showed elevation of urinary CD80. Reiser *et al*. reported that nephrin or α3 integrin knockout mice showed expression of CD80 on podocytes; they hypothesized that various stresses, including genetic defects, might lead to podocyte CD80 induction.

Our study showed that urinary CD80 is not affected by serum CD80 (Fig. 3). Importantly, we examined the correlation between serum and urine CD80 levels; we found that serum CD80 was within the reference range, even for serum samples from patients that simultaneously exhibited very high urinary CD80 values, suggesting that urinary CD80 is not derived from serum CD80.

We focused on the relationship between urinary protein and urinary CD80 and found a positive correlation (Fig. 2), which was observed in MCD in relapse, FSGS, and inherited NS (Supplemental Fig. 3). These results indicated that the elevation of urinary CD80 was not specific to patients with MCD in relapse, and it was correlated with urinary protein levels that might rather reflect the severity of glomerular damage, i.e., podocyte injuries. In contrast, in some samples, the correlation between urinary protein and CD80 was poor; this suggests that other factors may influence urinary CD80 excretion.

The current study has limitations. We did not perform Western blotting analysis of urinary CD80 or measure CD80 expression in kidney tissue. When examining urinary CD80, it is important to determine whether that urinary CD80 is blood-derived or kidney-derived; it is an important limitation that we did not perform these analyses. However, the method for measuring CD80 in renal tissues has been reported as low quality^15,16^; moreover, we showed that serum CD80 levels in our patients were not correlated with urinary CD80. The second limitation is that the sample sizes remain small in our study. The number of patients with FSGS or inherited NS was only four; however, 50% or 25% of these patients showed much higher levels of urinary CD80. Therefore, we judged that urinary CD80 might not be a useful diagnostic marker for renal diseases, and we decided not to collect more samples.

In conclusion, our results showed that urinary CD80 was not a reliable differential diagnostic marker between MCD in relapse and FSGS or other kidney diseases, including inherited kidney diseases, in contrast to previous reports. The level of CD80 was elevated in all patients with active kidney disease. We suggest that measuring urinary CD80 levels is not a reliable marker for the differential diagnosis of MCD in relapse and other kidney diseases.

## Methods

### Ethical considerations

All procedures were reviewed and approved by the Institutional Review Board of Kobe University Graduate School of Medicine (No_1770), with all study subjects providing written informed consent. All methods were performed in accordance with the relevant guidelines and regulations.

### Patients

We retrospectively analysed 30 subjects in the healthy control group (Cat. No. OH2000, OH2010, Sunfco Ltd., Tokyo, Japan) and 65 samples from 55 patients with MCD (n = 31), FSGS (n = 4), inherited NS (n = 4), Alport syndrome (n = 5), and other glomerular diseases (n = 11), including Henoch-Schönlein Purpura Nephritis (HSPN), IgA Nephropathy (IgAN), Hemolytic Uremic Syndrome (HUS), and oligomeganephronia (Table 1). Renal biopsies were performed on all patients with MCD, FSGS, Alport syndrome, and other glomerular diseases except for the case with HUS. These patients were diagnosed using established criteria^24–28^. Pathogenic mutations were proven in all patients of inherited NS (*NPHS1* p.Pro368Ser and p.Gln839Argfs*8, Ref:NM_004646, compound heterozygote) (*WT1* p.Cys393Tyr, Ref:NM_024426, heterozygote) (*ADCK4* p.Ser246Asn, Ref:NM_024876, homozygote) (*ACTN4* p.Leu224Pro, Ref:NM_004924, heterozygote) and Alport syndrome by genetic tests. Thirty-one patients with MCD provided 41 urine samples, including 10 patients who provided samples at both relapse and remission. They were divided into two groups, including a group in remission (n = 17), whose urinary protein creatinine ratio was <2.0 g/gCre, and a group in relapse (n = 24), whose urinary creatinine ratio was >2.0 g/gCre. Immunosuppressive therapy was introduced for all patients with MCD in relapse, or FSGS, and for some patients with IgAN and HSPN. This led to a reduction in proteinuria for all treated patients.

### CD80 measurement

The value of CD80 were measured by using urine and serum samples that had been stored at -80 °C. The sandwich ELISA method was used for measurement of urinary CD80. Nunc-Immuno module 96-well plates (Cat. No. 468667, Thermo Fisher Scientific, Waltham, MA, USA) were coated with 100 µl capture anti-CD80 antibody (Cat. No. 11-221-C100, Exbio, Vestec, Czech Republic). After the plates were incubated at 4 °C overnight for adhesion, the coating solution was removed completely. The coated wells were blocked with 300 µl of 1% BSA per well at 4 °C overnight. The urine and serum samples, standards of CD80 protein (Cat. No. 10698-H08H, Sino Biological Inc., Peking, China), and anti-CD80 antibody labelled with HRP (Cat. No. 37711 MAB140, R&D Systems Inc., Minneapolis, MN, USA) were diluted in PBS containing 1% BSA and were applied to wells with room temperature incubation for 90 min, followed by washing three times with PBS containing 0.05% Tween 20. The chemiluminescent substrate (Cat. No 37070, Thermo Fisher Scientific) was added and incubated for 15 min; then, absorbance values were read by using a microplate reader (FLUOstar OPTIMA, BMG Labtech).

### Statistical analysis

All results were described as median with interquartile range. Kruskal-Wallis and Dunn’s multiple comparison tests were used for analyses of differences among groups. The Mann-Whitney U test was used for comparisons between high and low CD80 groups in each renal disease, and for comparison of urinary CD80 between MCD in relapse and renal diseases other than MCD. Spearman rank correlation coefficient was used to calculate the correlation between urinary protein and urinary CD80. The level of statistical significance was set as p < 0.05. Data graphics and statistical analysis were described with GraphPad Prism7 software.

## Electronic supplementary material

Supplementary Dataset 1

## Data Availability

The datasets generated during and/or analysed during the current study are available from the corresponding author on reasonable request.
