# Supplementary material for: The utility of urinary CD80 as a diagnostic marker in patients with renal diseases
Source: Sci Rep. 2018 Nov 23;8:17322. doi: 10.1038/s41598-018-35798-2 (PMC6251900; doi:10.1038/s41598-018-35798-2)
Supplement: Supplementary file 1 — Supplementary Dataset 1 [file 41598_2018_35798_MOESM1_ESM.docx]

**Supplemental Information**

**The utility of urinary CD80 as a diagnostic marker in patients with renal diseases**

Shogo Minamikawa^1^, Kandai Nozu^1,^*, Shingo Maeta^2^, Tomohiko Yamamura^1^, Keita Nakanishi^1^, Junya Fujimura^1^, Tomoko Horinouchi^1^, China Nagano^1^, Nana Sakakibara^1^, Hiroaki Nagase^1^, Hideaki Shima^2^, Kenta Noda^2^, Takeshi Ninchoji^1^, Hiroshi Kaito^1^, Kazumoto Iijima^1^

*^1^Department of Pediatrics, Kobe University Graduate School of Medicine, Kobe, Japan*

*^2^Sysmex Corporation, Technology Development, Elemental Technology Development 2, Kobe, Japan*

***Corresponding author**

Kandai Nozu

Department of Pediatrics, Kobe University Graduate School of Medicine, 7-5-1 Kusunoki-cho, Chuo, Kobe, Hyogo 650-0017, Japan

Phone: +81-78-382-6090

Fax: +81-78-382-6099

E-mail: nozu@med.kobe-u.ac.jp

**Supplemental Fig. 1. Correlation between optical density (O.D.) and amount of CD80 (ng/ml) by our method and by established ELISA.**

Established method

Our method

A linear correlation was observed in both methods (our method: r = 1.0, p = 0.0004) (Established method: r = 0.96, p = 0.0028).

**Supplemental Fig. 2. Correlation of urinary CD80 between patients with MCD in relapse and patients with all renal diseases other than MCD.**

There was no correlation of urinary CD80 between MCD in relapse and all other renal diseases. This suggests that elevated urinary CD80 is found in patients with MCD in relapse, as well as patients with other renal diseases.

**Supplemental Fig. 3. Correlation between the difference in urinary CD80 and urinary protein in each renal disease.**

＊

＊

We calculated the 97.5th percentile for urinary CD80 level of the control group and determined the normal range of urinary CD80 is below 105.1 ng/gCr. Values greater than the 97.5th percentile of the control were defined as high CD80 levels. The remaining subjects in each group were defined as exhibiting normal CD80. We compared the amount of urinary protein between these two groups, according to each renal disease. There were significant differences between high and low groups for MCD in relapse and all renal diseases combined (*p < 0.05). Although the same tendencies were observed between FSGS and inherited NS, a significant difference was not detected because of the small sample size.
